# A descriptor-free machine learning framework to improve antigen discovery for bacterial pathogens

**DOI:** 10.1371/journal.pone.0323895

**Published:** 2025-06-05

**Authors:** Marco Podda, Castrense Savojardo, Pier Luigi Martelli, Rita Casadio, Alina Sîrbu, Corrado Priami, Alessandro Brozzi

**Affiliations:** 1 Department of Computer Science, University of Pisa, Largo Bruno Pontecorvo, 3, Pisa, Italy; 2 Dipartimento di Farmacia e Biotecnologie, Università di Bologna, Via Belmeloro, 6, Bologna, Italy; 3 GSK Vaccines, Via Fiorentina, 1, Siena, Italy; Chung-Ang University, KOREA, REPUBLIC OF

## Abstract

Identifying protective antigens (PAs), i.e., targets for bacterial vaccines, is challenging as conducting *in-vivo* tests at the proteome scale is impractical. Reverse Vaccinology (RV) aids in narrowing down the pool of candidates through computational screening of proteomes. Within RV, one prominent approach is to train Machine Learning (ML) models to classify PAs. These models can be used to predict unseen protein sequences and assist researchers in selecting promising candidates. Traditionally, proteins are fed into these models as vectors of biological and physico-chemical descriptors derived from their residue sequences. However, this method relies on multiple third-party software packages, which may be unreliable, difficult to use, or no longer maintained. Furthermore, selecting descriptors is susceptible to biases. Hence, Protein Sequence Embeddings (PSEs)—high-dimensional vectorial representations of protein sequences obtained from pretrained deep neural networks—have emerged as an alternative to descriptors, offering data-driven feature extraction and a streamlined computational pipeline. We introduce PSEs as a descriptor-free representation of protein sequences for ML in RV. We conducted a thorough comparison of PSE-based and descriptor-based pipelines for PA classification across 10 bacterial species evaluated independently. Our results show that the PSE-based pipeline, which leverages the FAIR ESM-2 protein language model, outperformed the descriptor-based pipeline in 9 out of 10 species, with a mean Area Under the Receiver Operating Characteristics curve (AUROC) of 0.875 versus 0.855. Additionally, it achieved superior performance on the iBPA benchmark (0.86 AUROC vs. 0.82) compared to other methods in the literature. Lastly, we applied the pipeline to rank unseen proteomes based on protective potential to guide candidate selection for pre-clinical testing. Compared to the standard RV practice of ranking candidates according to their biological descriptors, our approach reduces the number of pre-clinical tests needed to identify PAs by up to 83% on average.

## Introduction

The development of sub-unit protein vaccines against bacterial pathogens begins with identifying potential candidates for subsequent pre-clinical trials, where their ability to elicit a protective immune response in animal models is evaluated [1]. Proteins that successfully pass pre-clinical trials are termed protective antigens (PAs). This early phase is crucial for the entire vaccine development process, since a strong protective potential significantly increases the likelihood of successful optimization and downstream development, even if challenges in stability or formulation arise – challenges that later stages are specifically designed to address.

A significant challenge in pre-clinical testing is the limited capacity of laboratories to conduct *in-vivo* experiments. Due to the time- and cost-intensive nature of pre-clinical tests, researchers are compelled to refine lists of promising candidates to avoid exceeding a predefined resource budget.

The capacity for pre-clinical testing varies based on several site-specific factors. These are arduous to summarize, but can be estimated in the order of the hundred proteins per laboratory at most. A retrospective analysis of large bacterial PA discovery projects (Table 1) reveals that the median number of proteins tested by pre-clinical laboratories is 230. This number covers only a small fraction of the typical bacterial proteome, which usually comprises thousands of proteins. For instance, the proteome of *Neisseria meningitidis* (strain MC58) includes 2000 proteins. Yet, even the most extensive PA discovery project to date [2] could only manage to test a mere 350 (18%). Moreover, the current trend leans towards testing fewer candidates against a broader range of bacterial strains to ensure comprehensive coverage of circulating variants. As a result, the number of unique candidates progressing to the pre-clinical phase has decreased compared to the past.

**Table 1:** Literature review of past bacterial antigen discovery projects.

Species	Reference	Tested proteins	Antigens found
*Neisseria meningitidis*	[2]	350	28
*Streptococcus agalactiae*	[3]	312	4
*Shigella*	[4]	234	6
*Escherichia coli*	[5]	230	9
*Streptococcus pneumoniae*	[6]	108	6
*Streptococcus pyogenes*	[7]	52	1
*Streptococcus pyogenes*	[8]	40	6

In addition to the limitations imposed by pre-clinical capacities, identifying PAs is inherently challenging due to their small proportion within a bacterial proteome. This stark disparity is evident in PA discovery projects that have ambitiously attempted to test a large number of proteins (e.g., *Neisseria meningitidis* and *Streptococcus agalactiae* in Table 1). Consequently, discovering novel PAs can be considered as a “needle in a haystack” problem, further complicated by the stringent resource constraints dictated by pre-clinical capacities.

In response to these major challenges, Reverse Vaccinology (RV) [9] has emerged as a leading bioinformatics pipeline for identifying candidates from bacterial proteomes, with the goal of advancing to pre-clinical testing only proteins with strong vaccine potential. With respect to traditional vaccine development, RV offers significant ethical advantages by reducing reliance on animal testing and expediting the identification of viable candidates, in adherence to the R3 principles of ethical research – Replacement, Reduction, and Refinement [64]. Furthermore, RV enables faster and more targeted vaccine design, ensuring rapid responses to emerging infectious diseases. This not only aligns with the principles of ethical research but also enhances global health preparedness by streamlining the early stages of vaccine development.

Central to RV is the understanding that not all proteins are equally suitable as vaccine candidates, and that it is possible to identify promising ones by analyzing their residue sequences. To carry out the analysis, the wealth of information contained in the protein sequences is routinely distilled into a collection of biological or physico-chemical descriptors extracted with various bioinformatics tools.

Biological descriptors include, but are not limited to, the probability of being surface exposed, the likelihood of being an adhesin, and the predicted number of transmembrane domains. Physico-chemical descriptors, on the other hand, measure local residue properties such as counts, weight, and charge, as well as properties at the sequence level. Once these descriptors are extracted, they are used to identify promising candidates, either directly by filtering out proteins that fail to meet certain criteria (for instance, proteins with a low probability of being surface exposed), or indirectly by employing Machine Learning (ML) models trained to predict the likelihood of being PAs from the protein sequences represented as vectors of descriptors. Over the past two decades, experimental and data scientists from around the world have meticulously refined their unique “recipe” of descriptors for representing protein sequences (refer to [10] for an extensive review). Accordingly, a remarkable stream of bioinformatics tools for descriptor extraction have been developed [11–15].

Recent advancements in ML, particularly in the sub-field of Deep Learning [16], have introduced an innovative method for representing biological sequences, known as Protein Sequence Embeddings (PSEs). A PSE is a high-dimensional representation of a protein sequence, learned by training deep neural networks with self-supervision on large protein databases [17]. Typically, a PSE consists of one distinct embedding vector for each amino acid in the sequence. These vectors are contextualized, meaning that their values are shaped by the local and global context of the sequence in which the corresponding amino acid is situated. This allows for capturing the specific roles and interactions of each residue within its unique sequence context. The patterns encoded in PSEs are highly general, enabling their adaptation to learn a variety of predictive tasks, ultimately bypassing the necessity of a comprehensive understanding of the underlying physical or biological mechanisms [18–20].

Compared to conventional descriptors, PSEs are appealing because they facilitate the automatic extraction of features guided solely by the downstream predictive task, without requiring substantial domain expertise. Furthermore, they streamline the computational pipeline for converting protein sequences to vectors, merely requiring the weights of a trained neural network to calculate the embeddings. In contrast, extracting descriptors requires a multitude of bioinformatics software which can occasionally be defective (due to coding errors), challenging to acquire (for instance, due to non-permissive licensing), or become obsolete (i.e., their development or debugging is discontinued). However, despite their potential, PSEs have not been adopted by the RV community to date.

**Fig 1:**
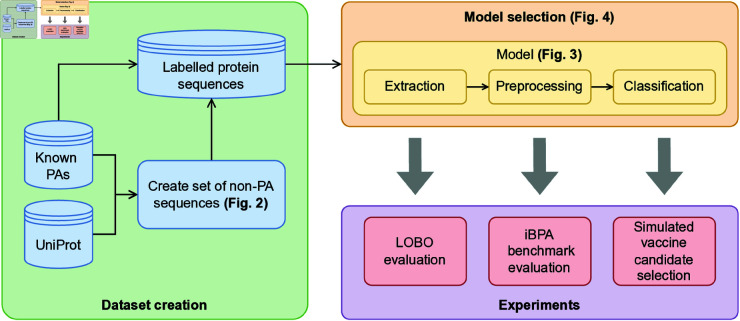
Overall workflow of this study. This work is structured in three stages. In the first stage (green box) we created a suitable dataset of PA and non-PA sequences. In the second stage (orange box) we selected a predictive pipeline to classify the sequences. In the third stage (purple box) we assessed the performances of the pipeline with three different experiments.

With these motivations, we developed a descriptor-free ML pipeline that takes raw FASTA sequences as input, and returns their probability of being PAs as output. The main component of the pipeline is a ML classifier that learns to assign high probability to known PAs and low probability to non-PAs. The crucial difference between the proposed pipeline and previous approaches is representing protein sequences as PSEs rather than as descriptor vectors. We compared the performance of the proposed pipeline with a similar descriptor-based pipeline using a dataset of protein sequences labeled as PAs or non-PAs. We independently tested the generalization capabilities of both methods using a Leave-One-Bacteria-Out (LOBO) validation strategy which consists of holding out the proteins of one bacterial species for testing, mimicking a scenario where a novel vaccine is sought for a previously unstudied bacterium. Our experimental results reveal that the proposed PSE-based pipeline outperformed the descriptor-based pipeline for 9 species out of 10 total, with an average Area Under the Receiver Operating Characteristics curve (AUROC) of 0.876 against 0.856. For a comprehensive comparison with other approaches in the literature, we also evaluated our pipeline on the iBPA benchmark (refer to [21] for further information), where it achieved the highest performance on all four metrics, with an AUROC of 0.86 (previously 0.82). Lastly, we show an application of the pipeline to improve the selection of candidates for pre-clinical testing. Specifically, we used our pipeline to rank the candidates in descending order according to the probability of being PA as assigned by our trained PSE-based pipeline. We compared the proposed ranking with a RV-based ranking determined on the values of biological descriptors. In repeated simulations using different bacterial proteomes, we show that testing candidates in the order proposed by our method leads to the re-discovery of known PAs with 83% less pre-clinical tests on average. A visual high-level summary of this work is provided in Fig 1.

The paper is structured as follows. The Background section introduces background concepts and discusses related work, while the Materials and methods section presents the computational methods used in the study. The Experiments section describes the experiments performed, while the Results section presents the results. Finally, the Discussion section discuses the significance of the results, the current limitations of the proposed approach, and the future research it might foster.

## Background

### Descriptors

As hinted in the introduction, descriptors are numerical values that quantify several characteristics (either biological or physico-chemical) of a protein sequence. Below, we review the two major categories of descriptors.

#### Biological descriptors.

Biological descriptors of amino acid sequences refer to attributes that relate to the biological function of the protein, such as motifs and patterns, post-translational modifications, and subcellular localization. Among biological descriptors, as claimed in the pioneering publication of RV [9], surface exposure—i.e., accessibility to antibodies—is the cornerstone criterion to be met by any vaccine candidate, either viral or bacterial. The tenet that vaccine antigens are outer proteins has been substantiated experimentally [8,22], and in a recent review, a statistical over-representation of antigens among extracellular proteins has been documented [21]. Additional evidence of the strong association between antigenicity and surface exposure can be found in several other works [2,23,24]. Accordingly, all the different flavors of RV available so far have subcellular localization as the primary filter [10]. Finally, in [25], surface exposure was identified (through a feature selection process among hundreds) as the driving feature to discriminate between antigens and non-antigens. Other commonly considered biological descriptors in RV include the presence of epitopes, the probability of being an adhesin, the number of transmembrane helices, and different immunogenicity scores. Nevertheless, besides surface exposure, there is no strong consensus about which precise set of biological descriptors makes a protein a “good” antigen. This might be due to the fact that the biological processes underlying our immunological response are still not completely understood as of today.

#### Physico-chemical descriptors.

Physico-chemical descriptors are a class of descriptors that encapsulate both global and local measurements, capturing a wide array of physical and chemical properties inherent in the sequences. One subset of these descriptors, known as compositional descriptors, quantifies specific attributes such as the hydrophobicity, charge, and molecular weight associated with individual amino acids within the sequence [26]. In addition to these, there are other commonly used descriptors that provide a more global perspective. These measure how the compositional descriptors are distributed throughout the sequence or how they change along the sequence. Auto-correlation [27] is a prime example of such a descriptor, providing a measure of the correlation between values at different points in the sequence. To capture more complex patterns that go beyond those expressed by single amino acids, these descriptors are often extended to consider di- and tri-peptides of the sequence. This allows to describe patterns and properties that emerge from the interactions and relationships between adjacent amino acids in the sequence, providing a more comprehensive representation of the sequence’s physico-chemical properties.

### Protein sequence embeddings.

Protein sequence embeddings are a type of representation for proteins that embodies the biological information inherent in the sequence. They are inspired by techniques in natural language processing, particularly word embeddings [28], which represent words as vectors in a way that captures semantic relationships between them.

Protein sequence embeddings are derived from protein language models (PLMs). These are essentially deep neural networks trained on vast collections of protein sequences by minimizing a self-supervised objective known as masked language modeling [17]. In simple terms, the model is tasked with predicting amino acids in the sequence that have been intentionally hidden or “masked,” forcing the model to learn from the context in which the missing amino acid appears. This approach is similar to the original training of word embeddings, where a word is predicted based on its context within a sentence. As a result of this process, each amino acid in the sequence is represented as a high-dimensional vector.

Each entry in the vector can be thought of as a feature that captures some aspect of the protein’s biological or physico-chemical properties. The exact interpretation of these features can be complex, as they are learned by the model and not directly tied to specific, predefined characteristics. However, the key is that proteins (or amino acids) that are similar in some biological sense will have similar embeddings. PSEs have been shown to encode various hallmarks of biological sequences, ranging from simple physico-chemical properties to more complex ones such as remote homology [29]. Thus, they have been used to learn a variety of sequence-based predictive tasks [18–20], although their use for antigen prediction has not been explored yet.

### Related work

In the past years, several RV methods to identify potential PAs out of bacterial proteomes have been proposed. They can be roughly categorized into filtering approaches and ML approaches.

Filtering approaches such as NERVE [30], Vaxign [31], Jenner-predict [32], VacSol [33], work by applying filters to the candidates until a viable subset is found. These filters are typically based on applying a cut-off value to biological or physico-chemical descriptors. Multiple filters are applied sequentially, in no particular order among themselves. For example, one could decide to exclude (from an initial set of candidates) protein sequences with predicted probability of being an adhesin below 0.5. On the remaining set, one could further decide to exclude those with predicted number of transmembrane domains below 2, and so on.

Traditional ML approaches such as VaxiJen [34], the methods by Heinson [35] and Bowman [25], VaxignML [21], and and deep learning-based approaches such as Vaxi-DL [36], Vaxign-DL [37] learn a model that classifies protein sequences as PAs or non-PAs from a labeled dataset of protein sequences. Crucially, in contrast with filtering approaches, these methods do not discard candidates, but rather assign a score (generally a probability) that indicates the likelihood of being PAs. Typically, trained ML models are applied to the entire proteome of a bacterial species for which a vaccine is sought. The proteins can be ranked according to the probability assigned by the model, indicating which ones are more likely to be protective.

In this work, we set our focus on this second category. To the best of our knowledge, using PSEs to represent sequences in this applied context has not yet been explored by the RV community, which usually relies on representing protein sequences as vectors of descriptors.

## Materials and methods

### Data collection and preprocessing

We collected 708 unique PA sequences from databases Protegen [38] and VaxiJen [34] with a corresponding UniProt entry. We removed homologs by clustering the sequences with mmseqs-cluster [39] (sequence identity > 0.3, coverage > 0.5), taking one representative sequence for each cluster found. This narrowed down the number of PA sequences to 458 (comprising 82 different bacterial species).

**Fig 2:**
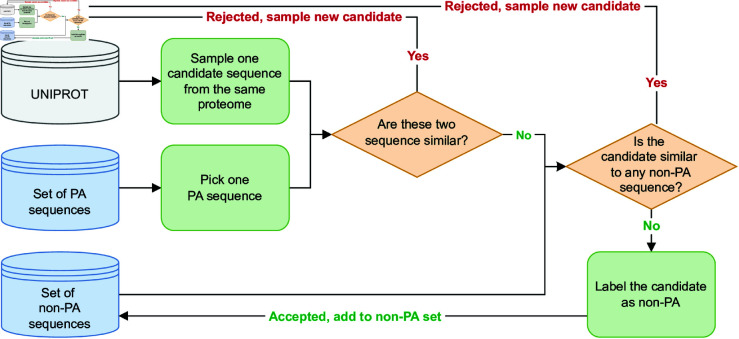
Flowchart of the non-PA sequences selection process. Given a reference PA sequence, a non-PA candidate sequence is drawn at random from the reference’s proteome in UniProt. The two sequences are checked for similarity: if they are similar, the candidate is rejected and a new one is drawn. If they are not, the candidate is tested for similarity against all previously accepted non-PAs. If they are, the candidate is rejected and a new one is drawn. Otherwise, the candidate is added to the set of non-PA sequences. This process is repeated for each PA sequence available.

While Protegen and VaxiJen only contain PA sequences, ground truth non-PA sequences are not available for this predictive task. Therefore, we artificially created non-PA samples following previous works [21,35]. The process to add a protein sequence to the set of non-PA sequences is exemplified in Fig 2. For a given PA sequence, we randomly sampled from UniProt one candidate non-PA sequence of the same bacterial species. We rejected the candidate if found to be similar to any PA sequence in the set of antigenic sequences or to non-PA sequences already accepted (sequence identity > 0.3, coverage > 0.5, *e*-value < 0.001). Otherwise, we accepted the candidate.

We finally assigned to each PA sequence a positive label (1), and to each non-PA sequence a negative label (0). In total, the constructed dataset contains 916 labeled sequences.

### Computational pipeline

**Fig 3:**
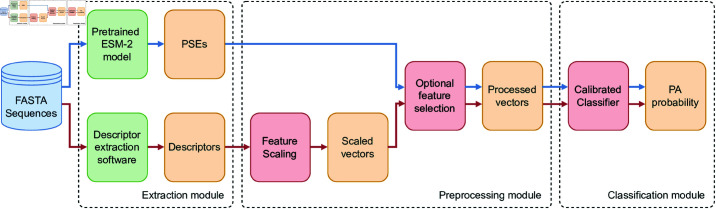
Overview of the computational pipeline. The pipeline proposed in this study is composed of three modules, which vary depending on how protein sequences are represented (blue path: PSE-based; red path: descriptor-based). Green boxes represent fixed operations that are applied once to the sequences, while red boxes are tuned during the experiments, meaning that their configuration is chosen during model selection to maximize performances. Orange boxes indicate numerical quantities, either vectors or scalars.

The core object of this study is a predictive pipeline that accepts protein sequences in FASTA format as input and yields a probability value $\hat{y}\in (0,1)$ for each protein sequence, indicating their likelihood of being a PA. Irrespective of the numerical representation of protein sequences (be it PSEs or descriptors), the pipeline can be deconstructed into three distinct sub-modules. The initial module, referred to as the Extraction module, processes protein sequences in FASTA format and converts them into numerical vectors with d∈ℕ components, depending on whether a PSE-based or descriptor-based representation is chosen. The subsequent module, known as the Preprocessing module, applies various transformations to the vectors, such as scaling and optional feature selection. The final module, termed the Classification module, accepts the preprocessed vectors and inputs them into a calibrated classifier that generates the ultimate probabilistic predictions. Importantly, the exact configuration of the Preprocessing and Classification modules (e.g., the amount of feature selection, the specific classifier) is chosen during model selection to maximize performances. The pipeline is depicted in Fig 3. Notice that, once trained, the pipeline can be applied to predict the proteins of any bacterial species without retraining, regardless of whether the species is rare or under-studied.

In the remainder of this section, we discuss the specific modules in detail in relation to the protein representation of choice (PSE- or descriptor-based).

#### Extraction module.

The extraction module of the descriptor-based pipeline consists of a stack of bioinformatics software which are used to extract both biological and physico-chemical descriptors. For a given input amino acid sequence, we collected *d* = 1560 descriptors from 6 different bioinformatics software:

- 6 biological descriptors pertaining subcellular localization obtained from the PSortB software package [11];
- 1 biological descriptor for the probability of being an adhesin, obtained from the SPAAN software package [12];
- 1 biological descriptor for the probability of containing signal peptides, obtained from the SignalP 4.1 software package [40];
- 4 biological descriptors obtained from the TMHMM 2.0 software package [13], regarding the number and composition of transmembrane helices;
- 1 biological descriptor corresponding to the immunogenicity score provided by the IEDB software package[14];
- 1547 physico-chemical descriptors obtained from the ProPy software package [41], including amino acid composition, auto-correlation, and quasi ordered sequence numbers.

The Extraction module of the PSE-based pipeline utilizes a publicly accessible, pre-trained PLM from Facebook Artificial Intelligence Research (FAIR), known as ESM-2 (https://github.com/facebookresearch/esm). This model is a deep transformer architecture comprising 33 hidden layers, pretrained on the UniRef-50 dataset using a masked language modeling approach. After a preliminary comparison with other models such as ProteinBERT [60] and ESM-1, ESM-2 emerged as the best candidate to serve as Extraction module. Our finding is aligned with previous results in the literature, where ESM-2 outperformed several single-sequence protein language models across a range of structure prediction tasks [55,59,62,63]. We fed the FASTA sequences to the model and collected the output from the $33^{\text{rd}}$ layer, which is the final hidden layer of the network. Given a single sequence, the output from this layer is a matrix of dimension L×d, where *L* denotes the sequence length and *d* = 1280 denotes the embedding dimension (i.e., there is one embedding for each amino acid in the sequence). By averaging the amino acid embeddings across the length dimension, we obtained a single PSE of size *d* = 1280 representing the entire sequence. S1 Fig shows the different processes that allow to obtain a vector of descriptors (left) and a PSE vector (right) from an example residue sequence.

#### Preprocessing module.

The first step of the Preprocessing module is a feature scaling step that transforms the features in a suitable range to avoid slow convergence of the downstream classifiers. During model selection, we opted between standard scaling, which independently scales all vector components in relation to the mean, and min-max scaling, which independently scales all components within the [0, 1] range. This step is exclusively applied to the descriptor vectors, as PSEs are already scaled appropriately.

The second step is a feature selection step accomplished with Principal Component Analysis (PCA). PCA is a technique that converts a set of potentially correlated features into a set of uncorrelated principal components via an orthogonal projection. It can be employed for dimensionality reduction by selecting the top-*k* principal components based on the original variance they preserve. During model selection, we determined whether to apply PCA and, if so, the number of components to retain. Alternatively, all features could be retained if PCA was not applied.

#### Classification module.

The Classification module consists of a classifier chosen along with its hyper-parameters during model selection from a pool of 5 alternatives:

- Logistic Regression (LR) [42] uses the logistic function to model the probability of the positive class. The coefficients of the logistic regression model are estimated by maximum likelihood.
- Random Forest (RF) [43] is an ensemble learning method that constructs a multitude of decision trees at training time and outputs the class that is the mode of the classes of the individual trees.
- eXtreme Gradient Boosting (XGB) [44] is a gradient boosting framework that uses a linear model solver and a sequence of decision trees as base models. At each iteration, the current model is trained to reduce the error committed by the previous.
- Support Vector Machine (SVM) [45] is a maximum margin classifier that operates by finding the hyperplane which maximizes the distance between the two classes. It can use the kernel trick to transform the input space, enabling it to handle non-linear classification boundaries.
- Multi-Layer Perceptron (MLP) [46] is a feedforward neural network with a single hidden layer and ReLU activations.

### Classifier calibration

While this work deals with a probabilistic binary classification task, not all the classifiers described above return proper probabilities. For example, the SVM predicts a score which is related to the distance of the input from the margin. To ensure that the classifiers output proper probabilities, we applied calibration to the models after training. Specifically, model calibration tries to enforce the following property on the outputs $\bar{y}$ of a classifier:


$$P(Y=1|\hat{Y}=\bar{y})\approx \bar{y},$$

where *P* indicates probability, *Y* is a discrete random variable which ranges in the set {0,1} of possible labels, and $\hat{Y}$ is a continuous random variable ranging in the interval [0,1]. In words, calibration consists of adjusting the classifier’s outputs so that they match the observed class frequencies. In this work, calibration is performed using Platt’s Scaling [47]. Basically, the method produces calibrated probabilities $\hat{y}$ with the following logistic regression model:


$$\hat{y}=\frac{1}{1+\exp(a\;\bar{y}+b)},$$

where parameters a,b∈ℝ are estimated with maximum likelihood.

### Model selection

During the evaluation process, we used randomized hyperparameter search [48] to optimize the pipeline steps. This included the scaling method (applicable only for the descriptor-based pipeline), the decision to apply feature selection and its extent, the choice of classifier and its hyper-parameters. Randomized hyper-parameter search entails sampling hyper-parameter combinations from predefined distributions and assessing the model performance with each combination. In this study, each hyper-parameter combination was evaluated using 5-fold Cross-Validation (CV). The combination that produced the best average negative log-likelihood (NLL) across the 5 validation folds was chosen. We used NLL to select the best pipeline since it has been shown to consistently drive the selection towards well-calibrated classifiers [49]. The resulting optimized pipeline was then trained on the complete training set and subsequently evaluated on a separate test set to obtain an unbiased performance estimate. The full table of the hyperparameters that were optimized during model selection is shown in S1 Table. Notice that in this study we do not take into account class imbalance, meaning that PAs and non-PAs are in the same proportion within the training set. The rationale of this choice is two-fold. On the one hand, previous studies in PA classification found that performances were not significantly impacted when the dataset is artificially imbalanced [21]. On the other hand, dealing with imbalanced data with over-sampling or under-sampling is known to negatively affect model calibration [61].

## Experiments

In this section, we provide the details of the three experiments carried out in this work. For each section describing an experiment, the first part describes the goal of the experiments and how the comparison with competing methods was structured, while the second part is devoted to describing the metrics used to measure performance. The data and code used in this study, as well as the experimental results, are available in the accompanying GitHub repository: https://github.com/marcopodda/dfpac. This includes all protein sequences used in this work, together with their vectorial representations (descriptors and PSE). Further information to reproduce the experiments can be found in the repository.

### PSE-based vs. descriptor-based model evaluation

**Fig 4:**
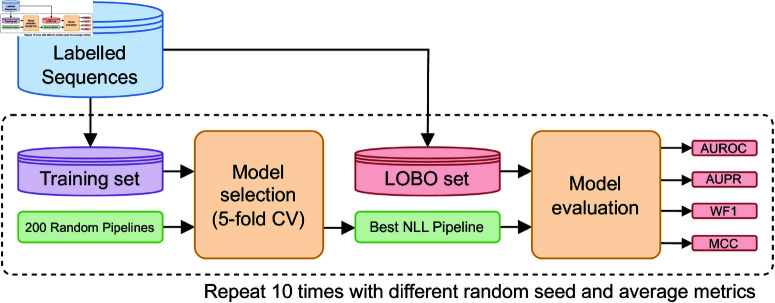
Leave-One-Bacteria-Out (LOBO) experimental setup. NLL: negative log-likelihood; AUROC: Area Under the Receiver Operating Characteristics curve; AUPR: Area Under the Precision-Recall curve; WF1: Weighted F1 score; MCC: Matthews Correlation Coefficient.

The initial experiment involved a comparative analysis of the descriptor-based pipeline and the PSE-based pipeline. The objective was to ascertain which data representation – descriptors or PSEs – offers superior predictive accuracy in identifying protective antigens. It is important to remark that we are *not* evaluating a single classifier, but the entire pipeline, which also includes the model selection of the classifier. We employ a Leave-One-Bacteria-Out (LOBO) evaluation scheme: for a given bacterial species, all protein sequences in the labeled dataset belonging to that species are allocated to the test set, while the remaining sequences are assigned to the model selection set. This approach tests the model’s ability to generalize to unseen bacterial species, aligning with methodologies used in prior studies [21,36,37] and to the typical RV stage of vaccine research and development projects in the pharmaceutical industry. We performed LOBO evaluations on 10 bacterial species (6 Gram positive, 4 Gram negative): *Actinobacillus pleuropneumoniae*, *Campylobacter jejuni*, *Chlamydia muridarum*, *Escherichia coli*, *Mycobacterium tuberculosis*, *Neisseria meningitidis*, *Staphylococcus aureus*, *Streptococcus pneumoniae*, *Streptococcus pyogenes*, and *Yersinia pestis*. Fig 4 shows the workflow of the chosen LOBO evaluation procedure.

**Metrics.** For each bacterial species tested, we used the trained model to predict its (unseen) protein sequences. We employed the following metrics for the comparison, similarly to previous works such as [21,36,37]: Area Under the Receiver Operating Characteristic (AUROC), Area Under the Precision-Recall curve (AUPR), Weighted F1 (WF1), and Matthews Correlation Coefficient (MCC). To smooth out the effect of randomness, the training procedure was repeated 10 times, each time using a different fixed seed for random numbers generation. We report the mean of each performance metric across these 10 trials. Once a random seed is fixed during a trial, the two performances (PSE-based vs. descriptor-based) are comparable fairly since they use the same data splits and undergo an identical model selection.

### Benchmark evaluation

In this experiment, we compare the PSE-based pipeline to methods from the literature on the iBPA benchmark [21]. The original iBPA benchmark includes 249 proteins, but we were only able to recover 243 upon request to the authors. To ensure a fair comparison, we trained the PSE-based pipeline using the same dataset as the reference work. Essentially, the training dataset for this task consisted of 397 antigens and 397 non-antigens. We excluded 3 proteins from the original dataset due to unclear or non-bacterial species. During training, the PSE-based pipeline was optimized with the same hyper-parameter tuning procedure as in the LOBO evaluation. However, we fixed the classifier to SVM since in the LOBO evaluation it performed better than the alternatives 71% of times on the validation set. We compared against 8 different methods from the literature, reusing the results from the work of [37]. We remark that the PSE-based pipeline is trained with 394 antigens out of the original 397 (99.2%), and tested on 243 proteins out of the original 249 (97.5%). We therefore consider the comparison fair to the best of our possibilities.

#### Metrics.

For the benchmark comparison, we utilized the same metrics as those employed in the LOBO evaluation. We report the mean of evaluating the PSE-based pipeline with 10 different random seeds, which is consistent with our approach in previous experiments.

### Candidate selection simulation

In this experiment, we simulate a situation where a researcher, provided with a bacterial proteome, must determine the order in which candidate antigens should proceed to pre-clinical tests (assuming a sequential testing protocol). The optimal approach would prioritize actual antigens for testing, thereby saving pre-clinical resources by minimizing tests on non-antigens. We compare two distinct strategies for establishing this testing sequence:

- one that ranks the candidates according to their biological descriptors, similar to the filtering approach used in RV, which we term the *RV-based* approach,
- and another that exploits the output probability given by the PSE-based pipeline trained during the LOBO evaluation to rank candidates, which we call the *likelihood-based* approach.

We downloaded the entire proteomes of the 10 test species mentioned above from UniProt. These proteomes were enriched by adding, for a given species, all PA sequences found in our dataset (taking care of removing duplicate sequences). Since surface exposure is the gold standard that all candidates must adhere to in order to be eligible, the comparison was restricted to protein sequences whose subcellular localization was predicted as “extracellular space” or “outer membrane” by the BUSCA software package [50]. After filtering for surface exposure, we assigned a positive label to known PAs (1), and a negative label (0) to the remaining proteins. Note that this approach is conservative, since proteins that we deem as negative are actually untested, and therefore it is unknown whether they are PA or not.

The RV filtering strategy was implemented by arranging the protein sequences using 4 descriptors as sorting keys: probability of having signal peptides (from high to low), probability of being an adhesin (from high to low), number of transmembrane helices (from large to small), immunogenicity score (from high to low). The order of precedence among the 4 keys was randomly determined in each trial (since there is no universal preference criteria among them). Conversely, the likelihood-based strategy was implemented by sorting the protein sequences in descending order according to their likelihood of being PAs. More precisely, given a bacterial species, the likelihoods were assigned from the corresponding PSE-based pipeline trained during the LOBO evaluation (meaning that the protein sequences of the bacterial species under study were never seen during training).

#### Metrics.

To compare performances in this experiment, we devised a novel metric designed to reward assigning higher rank to known PAs. Given a bacterial proteome composed of *m* protein sequences $S=(s^{\left(1\right)},\ldots ,s^{\left(m\right)})$, let $y_{S}=\langle y^{\left(1\right)},\ldots ,y^{\left(m\right)}\rangle$ be a vector that labels the sequences in *S* as known PAs (1) or non-PAs (0). Let π be the “ideal” permutation of $y_{S}$ that rearranges all known PAs first, and $y_{S}^{\pi }$ be the label vector rearranged accordingly. Similarly, let $\hat{\pi }$ be a permutation to evaluate, and $y_{S}^{\hat{\pi }}$ be the label vector rearranged accordingly. In practice, the permutation $\hat{\pi }$ is obtained by arranging the sequences in *S* according to some precomputed ranking (be it RV-based or likelihood-based). Finally, let $\phi :{\mathbb{R}}^{m}\to {\mathbb{R}}^{m}$ be a cumulative sum function defined over vectors as follows:

$$\phi (z)=\langle z_{1},z_{1}+z_{2},\ldots ,\sum_{i=1}^{m}z_{i}\rangle ,\;z\in {\mathbb{R}}^{m}.$$ (1)

**Fig 5:**
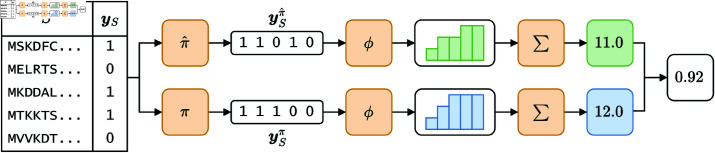
Example of the nADR metric computation. We employ a toy proteome composed of *m* = 5 protein sequences, of which 3 are known PAs. In the picture, ϕ transforms the input vector in a histogram of running sums, while ∑ sums the histogram bins.

Conceptually, the vector returned by ϕ is a histogram where the *i*-th bar represents the running sum up to the *i*-th element of the input vector. With these information, we can define the *normalized Antigen Discovery Rate* (nADR) as:

$$nADR(y_{S}^{\hat{\pi }},y_{S}^{\pi })=\frac{\sum_{i=1}^{m}\phi (y_{S}^{\hat{\pi }}{)}_{i}}{\sum_{i=1}^{m}\phi (y_{S}^{\pi }{)}_{i}},$$ (2)

where the *i* subscript is used to denote the *i*-th component of the vector returned by ϕ. In practice, the nADR first calculates the area of the two histograms (by summing up the histogram bins) and then computes the ratio of the two areas obtained. Intuitively, it quantifies how closely the evaluated ranking matches the ideal ranking, with a higher score indicating a better match, akin to information retrieval metrics like the normalized Discounted Cumulative Gain [51]. A simple illustration of how the metric is computed is shown in Fig 5. Note that nADR has many desirable characteristics: it ranges in the interval (0,1] since $\phi (y_{S}^{\hat{\pi }}{)}_{i}\leq \phi (y_{S}^{\pi }{)}_{i}$ holds trivially for 1≤i≤m; it is independent of *L* since it is normalized; and it equals 1 if and only if $\hat{\pi }=\pi$ (i.e., when the evaluated ranking is optional). It is not defined when the denominator is 0, i.e., when the proteome does not contain known PAs, in which case we simply force it to be 0. In our experiments, nADR is computed 10 times with different random seeds for each held-out bacterial species. We report the mean of these trials.

To further characterize the quality of the rankings, during the experiments we also monitor an index that we term the *First Hit Index* (FHI), defined as:

$$FHI(y_{S}^{\hat{\pi }})=\min\{i|(y_{S}^{\hat{\pi }}{)}_{i}=1,\;1\leq i\leq m\},$$ (3)

which stores the position of the first known antigen in ranking order. Intuitively, it can be interpreted as the minimum number of pre-clinical tests needed to recall a known antigen. Referring back to Fig 5, we have that $FHI(y_{S}^{\hat{\pi }})$ = 2.

### Fold enrichment analysis

To further characterize the results of the previous experiment, we conducted a fold enrichment analysis of known PAs within the 90-th percentile of the average (across 10 different random seeds) ranking produced by the likelihood-based strategy. In simpler terms, we checked the statistical over-representation of known PAs within the high-probability subset of the proteome. The fold enrichment is defined as the ratio between the observed number of PAs in the 90th percentile of the ranking and the expected number across the whole proteome. On the same subset, we computed the recall metric, defined as the percentage of known PAs found in the 90-th percentile. To add statistical significance to the results, we also computed the *p*-values (at the conventional 0.05 cutoff) associated to the enrichment with a hypergeometric test.

## Results

Here, we present the results of the experiments detailed previously. Each sub-section refers to an experiment, following the same order in which they are described in the Experiments section.

### PSEs outperform descriptors in LOBO evaluation

Table 2 summarizes the results of the evaluation as described in the Experiments section. In 7 out of 10 species (*A. pleuropneumoniae*, *C. jejuni*, *C. muridarum*, *E. coli*, *M. tuberculosis*, *N. meningitidis*, *S. pneumoniae*), the PSE-based pipeline outperforms the descriptor-based pipeline across all measured metrics. In two cases (*S. aureus* and *Y. pestis*), PSE-based performs better on 3 metrics out of 4. Only in one case (*S. pyogenes*), the descriptor-based pipeline performs better than the PSE-based baseline (in 3 metrics out of 4). Lower generalization of *S. pyogenes*’ PSEs with respect to descriptors might be caused by an underrepresentation of sequence characteristics or distributional properties in the sequences used to pretrain the ESM-2 model. More precisely, it could be that the *S. pyogenes* sequences, while antigenic, are different from all other sequences (perhaps due to peculiarities of the sequencing process). In that case, it might be that this variability has been overfitted by PSEs, while descriptors are able to better generalize. However, ours is merely a hypothesis that needs to be tested in further studies. Indeed, the gap between PSEs and descriptors, while in favor of the latter, is still narrower than the other cases. Also, the MCC metric, which measures correlation between the predictions and the actual antigenicity, is slightly in favor of PSEs.

**Table 2:** Leave-One-Bacteria-Out evaluation of descriptor-based and PSE-based pipelines.

Species (Gram)	Pipeline	AUROC	AUPR	WF1	MCC
*A. pleuropneumoniae* (–)	Descriptors	.958±.015	.966±.010	.858±.014	.743±.036
	PSEs	**.992**±.007	**.993**±.006	**.880**±.040	**.784**±.065
*C. jejuni* (–)	Descriptors	.940±.013	.945±.019	.839±.021	.685±.040
	PSEs	**.958**±.010	**.958**±.011	**.883**±.029	**.768**±.055
*C. muridarum* (–)	Descriptors	.829±.011	.870±.015	.769±.031	.566±.062
	PSEs	**.872**±.012	**.892**±.011	**.788**±.024	**.611**±.043
*E. coli* (–)	Descriptors	.918±.010	.893±.015	.853±.017	.708±.034
	PSEs	**.935**±.007	**.920**±.012	**.892**±.013	**.783**±.025
*M. tuberculosis* (+)	Descriptors	.750±.012	.804±.026	.658±.022	.424±.028
	PSEs	**.772**±.010	**.850**±.007	**.724**±.018	**.468**±.029
*N. meningitidis* (–)	Descriptors	.790±.012	.828±.012	.681±.028	.362±.057
	PSEs	**.832**±.015	**.866**±.035	**.777**±.010	**.554**±.019
*S. aureus* (+)	Descriptors	.932±.009	.966±.004	.836±.043	.667±.086
	PSEs	**.934**±.009	**.963**±.006	**.859**±.033	**.713**±.066
*S. pneumoniae* (+)	Descriptors	.790±.028	.815±.030	.667±.034	.341±.069
	PSEs	**.841**±.015	**.870**±.012	**.738**±.031	**.510**±.061
*S. pyogenes* (+)	Descriptors	.840±.013	.878±.009	.795±.013	.592±.028
	PSEs	**.812**±.012	**.808**±.017	**.791**±.025	**.599**±.052
*Y. pestis* (–)	Descriptors	.801±.048	.813±.046	.758±.046	.515±.095
	PSEs	**.805**±.017	**.718**±.010	**.790**±.029	**.586**±.053
Avg. Performance	Descriptors	.855±.017	.878±.019	.771±.027	.559±.054
	PSEs	**.875**±.011	**.884**±.013	**.812**±.025	**.638**±.047

Best results are shown in boldface. Gray overlays on the table cells are used to highlight cases where PSE-based pipelines performed better than descriptor-based pipelines. PSE: Protein Sequence Embeddings. AUROC: Area Under the Receiver Operating Characteristics curve. AUPR: Area Under the Precision-Recall curve. WF1: Weighted F1. MCC: Matthews Correlation Coefficient.

Averaging performances across species (last row of the table), we found that for all metrics, the PSE-based baseline performed better than descriptor-based models. This result provides clear evidence that PSEs are a more effective means to represent proteins for RV tasks, with superior performance in general.

### PSE-based pipeline outperforms competitors in iBPA benchmark

Table 3 reports the results of the benchmark evaluation on the iBPA dataset, as detailed in the Benchmark evaluation section. The PSE-based pipeline outperformed all the competitors in all the benchmark metrics. Notably, we report a 6.75% improvement in WF1 and a 11.8% improvement in MCC with respect to the previous best model. Summing up, while the previous experiment demonstrated that the PSE-based pipeline proposed in this study generalizes better than descriptor-based pipelines in a LOBO setting, this result demonstrates that it generalizes better than all other methods in the literature on unseen proteins, regardless of the bacterial species.

**Table 3:** Comparison of our PSE-based pipeline with existing works on the iBPA benchmark.

Method	Reference	AUROC	AUPR	WF1	MCC
PSE-based	This work	0.86	0.84	0.79	0.58
Vaxign-ML*	[21]	0.82	0.84	0.76	0.51
Vaxign-DL*	[37]	–	–	0.74	0.49
Vaxi-DL*	[36]	–	–	0.70	0.46
Heinson-Bowman*	[35]	–	–	0.68	0.37
VaxiJen*	[34]	–	–	0.66	0.32
Epitope-based*	[52]	–	–	0.62	0.24
Vaxign*	[53]	–	–	0.56	0.27
Antigenic*	[54]	–	–	0.49	-0.02

Symbol * indicates that the corresponding results were taken from [37], while symbol – indicates that the corresponding metric was not published in the reference work. AUROC: Area Under the Receiver Operating Characteristics curve. AUPR: Area Under the Precision-Recall curve. WF1: Weighted F1. MCC: Matthews Correlation Coefficient.

### Likelihood-based ranking outperforms RV-based ranking in simulated candidate selection

Table 4 shows the results of the simulated candidate selection for pre-clinical tests, where we compare the proposed likelihood-based approach against the RV-based approach described in the Candidate selection simulation section. In all the 10 test species under study, the likelihood-based strategy significantly improves on the nADR metric with respect to the baseline, by 49% on average. By inspecting the FHI, we can also observe that in 8 cases out of 10 (i.e., excluding *M. tuberculosis* and *Y. pestis*), using the likelihood-based strategy leads to the re-discovery of the first known PA within at most 4 pre-clinical trials on average, while the RV-based strategy, in the same cases, requires at least 6 and at most 41. Even in the most difficult cases of *Y. pestis* (resp. *M. tuberculosis*), our strategy requires 18 (resp. 12) pre-clinical tests to re-discover the first known PA, while the RV-based strategy requires 125 (resp. 38). Overall, our approach allows to save (on average across the 10 species) up to 83% of pre-clinical tests with respect to the RV-based strategy.

**Table 4:** Simulated candidate antigen selection for pre-clinical testing

Species (Gram)	Antigens/Exposed (%)	Method	nADR	FHI	Savings
*A. pleuropneumoniae* (–)	6/203 (3.0%)	RV-based	0.472	41	
		Likelihood	**0.741**	**2**	**95%**
*C. jejuni* (–)	5/173 (2.9%)	RV-based	0.589	15	
		Likelihood	**0.846**	**3**	**80%**
*C. muridarum* (–)	6/76 (7.9%)	RV-based	0.565	10	
		Likelihood	**0.884**	**3**	**70%**
*E. coli* (–)	19/484 (3.9%)	RV-based	0.547	22	
		Likelihood	**0.700**	**4**	**82%**
*M. tuberculosis* (+)	7/286 (2.4%)	RV-based	0.504	38	
		Likelihood	**0.825**	**12**	**68%**
*N. meningitidis* (–)	13/194 (6.7%)	RV-based	0.558	13	
		Likelihood	**0.788**	**3**	**77%**
*S. aureus* (+)	12/181 (6.6%)	RV-based	0.554	8	
		Likelihood	**0.709**	**2**	**75%**
*S. pneumoniae* (+)	6/94 (6.4%)	RV-based	0.497	18	
		Likelihood	**0.790**	**2**	**89%**
*S. pyogenes* (+)	19/101 (18.8%)	RV-based	0.506	6	
		Likelihood	**0.840**	**1**	**83%**
*Y. pestis* (–)	3/373 (0.8%)	RV-based	0.489	125	
		Likelihood	**0.628**	**18**	**86%**
Avg. performance	–	RV-based	0.528	30	
		Likelihood	**0.785**	**5**	**83%**

Best results are shown in boldface. RV-based: baseline. Likelihood: proposed strategy. Antigens/Exposed: the number of true antigens among the total number of surface exposed proteins. nADR: normalized Antigen Discovery Rate. FHI: First Hit Index. Savings: percentage of pre-clinical tests saved by replacing the RV-based strategy with the likelihood-based strategy.

**Fig 6:**
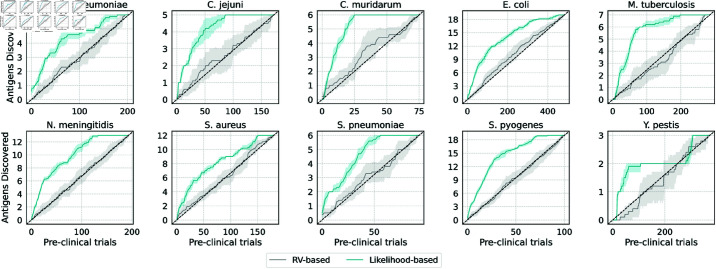
Comparison between likelihood-based strategy and RV-based strategy at discovering novel antigens (simulation). The plots display the number of pre-clinical trials (x-axis) in relation to the cumulative distribution of known PAs (y-axis) re-discovered with the proposed likelihood-based strategy (green line), compared to the RV-based strategy (gray line). Both strategies are described in the Candidate selection simulation section. Shading around the lines identify a 95% confidence interval around the mean of the cumulative distribution. In all cases, the green line is above the gray line, indicating that to re-discover the same number of known PAs, our method requires a smaller number of pre-clinical tests.

The evolution of the simulated pre-clinical tests is shown in Fig 6, where in the x-axis we indicate the number of pre-clinical trials (assuming they are performed sequentially), and on the y-axis we plot the cumulative distribution of known PAs re-discovered according to the likelihood-based strategy (green line) versus the RV-based strategy (gray line). As can be seen in all the species under study, the rate at which PAs are re-discovered with the likelihood-based approach is consistently above that of the RV-based approach, which indicates that to re-discover the same number of known PAs, the proposed method requires a smaller number of pre-clinical tests.

### High-probability subset of likelihood-based ranking is enriched with known PAs

The fold enrichment analysis, presented in Table 5, shows a statistically significant over-representation of known PAs within the 90th percentile of the ranking (i.e., the subset containing high-probable PAs according to the likelihood-based strategy) compared to the expectation under random sampling. The result is consistent across the 10 species evaluated, with *p*-values below the 0.05 cutoff. Remarkably, the proposed method is capable of recalling 36% of known PAs within the 90th percentile of the ranking (on average across the 10 species). These results suggest that the PSE-based pipeline is assigning known PAs to the top positions of the ranking at a higher rate than a random model, which conforms to our expectations.

**Table 5:** Fold enrichment analysis of Protective Antigens (PA) in the 90th percentile likelihood-based ranking.

Species	Size	90th Perc.	Exp.	Obs.	Enrichment	Recall	p -value
*A. pleuropneumoniae* (–)	203	21	0.62	2	3.22	33%	1.5e-2
*C. jejuni* (–)	173	18	0.52	2	3.84	40%	8.3e-3
*C. muridarum* (–)	76	8	0.63	2	3.17	33%	1.3e-2
*E. coli* (–)	484	49	1.92	7	3.64	37%	2.0e-4
*M. tuberculosis* (+)	286	29	0.71	3	4.23	43%	2.4e-3
*N. meningitidis* (–)	194	20	1.34	6	4.48	46%	4.9e-5
*S. aureus* (+)	181	19	1.26	4	3.18	33%	3.7e-3
*S. pneumoniae*(+)	94	10	0.64	2	3.13	33%	1.4e-2
*S. pyogenes* (+)	101	11	2.07	5	2.42	26%	5.2e-3
*Y. pestis* (–)	373	38	0.31	1	3.27	33%	2.8e-2

Notice that all *p*-values are below the standard 5.0e-2 cutoff for significance. Size: total population. 90th Perc.: size of the 90th percentile of the ranking. Exp.: expected number of known PAs Obs.: observed number of known PAs. Recall: the percentage of known PAs found in the 90th percentile of the ranking.

## Discussion

Since its inception two decades ago, research in RV has focused on skimming the number of potential good antigens to be tested in animals, searching for a signature of biological descriptors in the amino acid sequences. This extensive search has confirmed surface exposure as the unchallenged winner; in fact, descriptor-based ML attempts have found surface exposure as the major driver of discrimination between antigens and non-antigens, relegating other descriptors to marginal contributions. As such, many predictive tools are now currently available to reliably assess the subcellular localization of proteins. However, past antigen discovery projects have clearly shown that cellular localization is a necessary but not sufficient condition to establish whether a protein is a vaccine antigen.

With this motivation, the present study diverges from the longstanding paradigm for which there exists a “recipe” of biological descriptors which, in addition to surface exposure, can be used to identify good antigens. Instead, we explored the new emerging route of sequence embeddings, which has recently achieved breakthroughs in the field of biological sequence analysis. With our approach, the protein primary structure is used directly as input to the ML model, which learns a hidden discriminatory rule based on patterns of residue co-occurrences without imposing any *a-priori* knowledge (in the form of precomputed biological descriptors). Our experiments validate the hypothesis that being descriptor-agnostic can improve predictive performances.

A second major result presented in this paper is to show that ranking candidate antigens from top to bottom using a trained PSE-based model makes a better use of available resources (in this case, the budget imposed by pre-clinical capacities) than common strategies used by RV. In a sense, we shift the role of RV from “sieve” to a sort of “recommender”. Besides leading to a faster discovery of novel antigens as demonstrated experimentally, this new perspective allows to “broaden the horizon” by encouraging to test not-yet-studied proteins which are however ranked by the model in the top positions.

On a final note, we are also aware that the proposed approach has limitations. Firstly, both the descriptor-based pipeline and the candidate selection simulation are grounded on the accuracy of subcellular prediction methods, which, although reliable in general, are not flawless. Secondly, the classes of animal-tested PAs come from the stringency of experimenter-defined thresholds on laboratory read-outs which have a native numerical scale (like ELISA titers, human serum bactericidal assays titers, *p*-values in statistical analyses of challenge studies), which could generate noisy data. Thirdly, current PSE models cannot handle long protein sequences. In this study, we truncated the sequences at 1022 residues (with complete coverage for approximately 96% of the sequences in our dataset). In future works we would like address this issue once available methodologies to embed long sequences become mature [56–58]. Lastly, PSE are less interpretable than descriptors due to the fact the features that compose them are abstract and hierarchical, and do not map directly into biological insights.

All these aspects considered, the results presented in this study represent a relevant step towards improving the antigen discovery process, both in terms of reduction of animal tests, increased hit ratio of good antigens, and simplification of the computational discovery pipeline.

## Supporting information

S1 Fig Different processing of residue sequences. The figure shows how a residue sequence is processed to become a vector of descriptors (left) or a PSE (right), with the corresponding final dimensions (1560 for descriptors, 1280 for PSEs).
(TIFF)

S1 Table Table of hyperparameters. The table shows the different hyperparameters that were optimized during model selection.
(XLSX)
