# Supplementary figures and images for: A descriptor-free machine learning framework to improve antigen discovery for bacterial pathogens
Source: PLoS One. 2025 Jun 5;20(6):e0323895. doi: 10.1371/journal.pone.0323895 (PMC12140217; doi:10.1371/journal.pone.0323895)

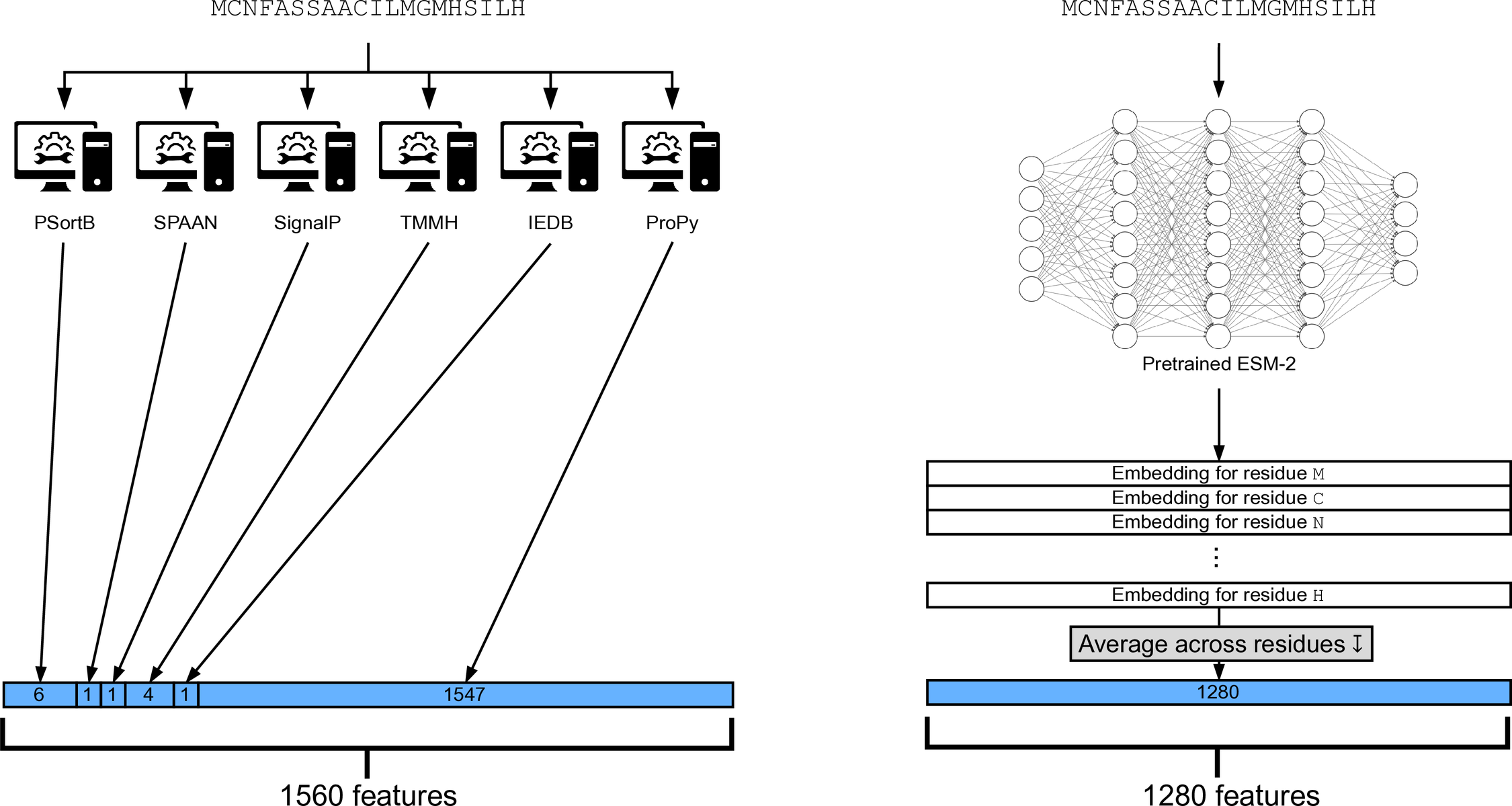

Supplement: S1 Fig — The figure shows how a residue sequence is processed to become a vector of descriptors (left) or a PSE (right), with the corresponding final dimensions (1560 for descriptors, 1280 for PSEs). (TIFF) [file pone.0323895.s001.tif]
